# Diagnosis of human granulocytic anaplasmosis in Belgium by combining molecular and serological methods

**DOI:** 10.1002/nmi2.65

**Published:** 2014-10-09

**Authors:** M Hing, S Woestyn, B Van Bosterhaut, Y Desbonnet, P Heyman, C Cochez, C Silaghi, H Sprong, P E Fournier, D Raoult, P Neirinckx, W Heuninckx

**Affiliations:** 1 Clinical Laboratory, National Reference Centre Anaplasma, Military Hospital Queen AstridBrussels, Belgium; 2 Laboratoire d'analyses médicales J. WoestynMouscron, Belgium; 3 Laboratoire de Biologie clinique, Centre Hospitalier de MouscronMouscron, Belgium; 4 General PractitionerMouscron, Belgium; 5 Research Laboratory for Vector-borne Diseases, Military Hospital Queen AstridBrussels, Belgium; 6 Comparative Tropical Medicine and Parasitology, Ludwig-Maximilians-Universität MünchenMunich, Germany; 7 Laboratory for Zoonoses and Environmental Microbiology, National Institute for Public Health and Environment (RIVM)Bilthoven, the Netherlands; 8 URMITE, UM63, CNRS 7278, IRD 198, INSERM 1095, Faculté de Médecine, Aix-Marseille UniversitéMarseille, France; 9 Military Hospital Queen AstridBrussels, Belgium

**Keywords:** *Anaplasma phagocytophilum*, anaplasmosis, human, laboratory diagnosis

## Abstract

We report here one new, hospitalized case of *Anaplasma phagocytophilum* in Belgium. The clinical presentation of anaplasmosis, its treatment and the molecular and serological relevant laboratory methods are briefly developed.

Dear Editor,

Human granulocytic anaplasmosis (HGA) is caused by the obligate intracellular bacterium *Anaplasma phagocytophilum*, which parasitizes neutrophilic granulocytes in mammalian hosts. This pathogen is transmitted by the bite of infected ticks of the family of Ixodidae. In Belgium a recent study detected *A. phagocytophilum* by PCR in 14% of the examined *Ixodes ricinus* ticks 1. The incubation period following the infecting tick bite is 5–21 days. Most patients have mild flu-like clinical signs but infection can result in hospitalization and severe complications have been described 2,3. The number of PCR-confirmed laboratory cases in Belgium and its neighbour countries France, Germany and the Netherlands is low, fewer than ten in the last 10 years 4–8.

A 60-year-old man without special medical history was seen by his general practitioner (GP) on 26 August 2013 with a fever of 40.5°C, chills, myalgia, arthralgia and severe headache of 3-day duration.

The patient noted the presence of a yellow-blue coloured bump in the groin area, possibly due to a tick bite, one week before symptom onset, while on holiday in the Ardennes, a forested region 100 km east of Brussels, the Belgian capital.

Initial laboratory results showed leucopenia (3.04 × 10^9^/L - reference values (rv) 4 × 10^9^ to 10 × 10^9^/L) with lymphocytopenia (8.9%), thrombocytopenia (93 × 10^9^/L; rv 150 × 10^9^ to 450 × 10^9^/L), moderately increased levels of transaminases (aspartate aminotransferase 46 U/L; rv <37 U/L; alanine aminotransferase 47 U/L; rv <40 U/L), lactate dehydrogenase (223 U/L; rv <193 U/L), and an increased level of C-reactive protein (131 mg/L; rv <5 mg/L) (Table1).

**Table 1:** Examination results of blood smears, PCR assays and IFA serology

Days after onset of symptoms	Blood smear results	PCR assay *msp2*	IgM (IFA)	IgG (IFA)
3	NF	Pos	<1:20	<1:64
6	NF	Pos	<1:20	<1:64
12	ND	Pos	1:2560	1:128
19	ND	Neg	1:640	1:256
26	ND	Neg	1:640	1:1024
33	ND	Neg	1:320	1:512
39	ND	ND	1:80	1:256
47	ND	ND	1:40	1:256
54	ND	ND	1:20	1:256
61	ND	ND	<1:20	1:256
89	ND	ND	<1:20	1:256

NF, not found of morulae within neutrophils cells; ND, not done; Pos, positive; Neg, negative; IFA, immunofluorescence assay; IgM <1/20, negative; IgG <1/64, negative.

As a result of the symptoms and the abnormal laboratory results, the GP referred this patient the same evening to the Emergency Department of a nearby Belgian hospital. The clinical examination showed no skin rash and no nausea.

All blood and cerebrospinal fluid (CSF) cultures were unrevealing and specific serological tests were negative. Blood and CSF samples were negative for antibodies to *Borrelia* spp. Serologies for hantaviruses, Epstein–Barr virus, cytomegalovirus, parvovirus and leptospires were also negative.

The patient was treated with ceftriaxone without amelioration of symptoms, with even an aggravation of his thrombocytopenia (37 × 10^9^/L; nl 150 × 10^9^ to 450 × 10^9^/L) on the fourth day of hospitalization. On the fifth day, addition of minocycline (100 mg twice a day for 1 week) resulted within 48 h in rapid improvement and recovery.

The Belgian National Reference Center for *Anaplasma* received serum and EDTA samples taken on 26 August and serum samples taken during the hospitalization. This reference laboratory is partially supported by the Belgian Ministry of Social Affairs through a fund within the Health Insurance System 9 and disposes of different diagnostic tools to confirm the diagnosis of anaplasmosis. The direct methods aim at visualizing the bacteria by microscopic detection of the characteristic intracytoplasmic inclusions (morulae) in the neutrophilic granulocytes and detecting specific nucleic acid sequences by real-time PCR. The indirect method detects the antibodies elicited by the infection by using an immunofluorescence assay (Focus Diagnostics, Cypress, CA, USA). This serology provides a retrospective diagnosis as IgM and IgG antibodies are not usually present during the early phase of the disease.

The microscopic examination of blood smears stained with May–Grünwald–Giemsa was negative.

The real-time PCR performed according to Courtney *et al*. 10 and targeting the *msp2 Anaplasma* gene detected the presence of *A. phagocytophilum* DNA in the acute phase (3rd day after the onset of symptoms) and was still positive on the 12th day. These two PCR-positive samples were subjected to an additional conventional PCR, targeting the *groEL* gene followed by DNA sequencing 11. Both samples yielded identical DNA sequences of 559 bp, which were 100% similar to four *A. phagocytophilum* isolates from GenBank. Two isolates from *Cervus elaphus* came from Spain (HM057223, HM057225) and two were from *I. ricinus* from Poland and Slovakia (KF312360, KF383239). Moreover, the sample from the 3rd day after the onset of symptoms was tested for *A. phagocytophilum* by using a conventional PCR assay that amplified a portion of the 16S rRNA gene followed by DNA sequencing. The primer pair used for both amplification and sequencing was 536F (5′-CAGCAGCCGCGGTAATAC-3′) and rp2 (5′-ACGGCTACCTTGTTACGACTT-3′). The PCR product was 987 bp, as expected, and its sequence was 99.6% identical to the 16S rRNA sequences from *A. phagocytophilum* strains Dog2 (CP006618), HZ (CP000235), HZ2 (CP006616), JM (CP006617), NE-16S-1 (JN990105), HB-SZ-HGA-S04 (HQ872464) and USG3 (AY055469). The obtained 16S rRNA sequence was deposited in GenBank under accession number KM 259921. All these findings unequivocally confirm the presence of *A. phagocytophilum* DNA in the patient samples.

The IgM antibodies to *A. phagocytophilum* peaked at the 12th day after onset of symptoms, the IgG antibodies peaked at the 26th day (Table1). This seroconversion with a fourfold rise in antibody titres, the PCR results and the epidemiological, clinical and biological criteria all confirm the diagnosis of HGA 2,12,13. We acknowledge the fact that as the majority of serological tests can still be negative early in the disease and no follow-up samples were examined for these pathogens, simultaneous infections with one of the other tested pathogens and *A. phagocytophilum* cannot be excluded.

In conclusion, we report a new case of human anaplasmosis in Belgium. This case highlights the importance of combining clinical suspicion with correct timely laboratory workup to diagnose and adequately treat a disease which is, at least in western Europe, rarely reported.
